# What Guidelines? Never Saw Them!

**DOI:** 10.1371/journal.pmed.0030413

**Published:** 2006-09-26

**Authors:** A. D Gowdy

Fretheim and colleagues' study confirms [1] yet again how difficult it is to change clinical practice. As a manager with a “big organization” National Health Service background, more recently working in practices with general practitioners (GPs), I always was impressed by GPs' shameless ability to ignore incoming paperwork, often to the extent of trashing the envelope unopened, especially the big brown ones from the local health authority.

I now work for a pharmaceutical company (not in my mind a competing interest, but I have declared it as such in the interests of transparency) where the ability to change practice is a key skill. When I compare the pharmaceutical company approach to that of the health authority, there are some key differences. It is easy to deride industry’s glossy advert approach, but this is simply the tip of the communications iceberg. In addition to the glossy adverts, there is immense attention by drug companies to detail. The key evidence-based messages are tested, honed, and polished and are then delivered via several channels repetitively. The channels include face-to-face delivery by the “detailers”. Progress is tracked meticulously.

This expensive but effective industry effort contrasts with the typical approach taken by the health authorities and other non-industry groups. The process of creating such guidelines has usually been so slow and difficult in the gestation that their credibility among GPs is low even before the rather dull photocopied paper is issued (by post, big brown envelope) and meets its predictable fate.

Fretheim and colleagues say “Key components were an educational outreach visit with audit and feedback, and computerized reminders linked to the medical record system. Pharmacists conducted the visits”. This feels a bit like a policing approach (another profession watching the GP) allied to computer direction, none of which feel particularly user-friendly. A non-audit nurse (or ex-drug-rep) calling briefly but frequently using the usual pens/mugs/Post-its reminder freebies might get a better response.

One study by Eve et al [2] did look at applying pharmaceutical company techniques to clinical change (“selling” Triple A therapy). It seemed to work. It may be worth bringing it back into focus.
